# DNA-Free Genetically Edited Grapevine and Apple Protoplast Using CRISPR/Cas9 Ribonucleoproteins

**DOI:** 10.3389/fpls.2016.01904

**Published:** 2016-12-20

**Authors:** Mickael Malnoy, Roberto Viola, Min-Hee Jung, Ok-Jae Koo, Seokjoong Kim, Jin-Soo Kim, Riccardo Velasco, Chidananda Nagamangala Kanchiswamy

**Affiliations:** ^1^Research and Innovation Centre, Genomics and Biology of Fruit Crop Department, Fondazione Edmund MachTrento, Italy; ^2^ToolGen Inc.Seoul, Republic of Korea; ^3^Center for Genome Engineering, Institute for Basic ScienceSeoul, Republic of Korea; ^4^Department of Chemistry, Seoul National UniversitySeoul, Republic of Korea

**Keywords:** genome editing, non-GMO, DNA-free, CRISPR/Cas9, apple, grapevine

## Abstract

The combined availability of whole genome sequences and genome editing tools is set to revolutionize the field of fruit biotechnology by enabling the introduction of targeted genetic changes with unprecedented control and accuracy, both to explore emergent phenotypes and to introduce new functionalities. Although plasmid-mediated delivery of genome editing components to plant cells is very efficient, it also presents some drawbacks, such as possible random integration of plasmid sequences in the host genome. Additionally, it may well be intercepted by current process-based GMO regulations, complicating the path to commercialization of improved varieties. Here, we explore direct delivery of purified CRISPR/Cas9 ribonucleoproteins (RNPs) to the protoplast of grape cultivar *Chardonnay* and apple cultivar such as *Golden delicious* fruit crop plants for efficient targeted mutagenesis. We targeted *MLO-7*, a susceptible gene in order to increase resistance to powdery mildew in grape cultivar and *DIPM-1, DIPM-2*, and *DIPM-4* in the apple to increase resistance to fire blight disease. Furthermore, efficient protoplast transformation, the molar ratio of Cas9 and sgRNAs were optimized for each grape and apple cultivar. The targeted mutagenesis insertion and deletion rate was analyzed using targeted deep sequencing. Our results demonstrate that direct delivery of CRISPR/Cas9 RNPs to the protoplast system enables targeted gene editing and paves the way to the generation of DNA-free genome edited grapevine and apple plants.

## Introduction

Grape and apple fruit crop plants are a major source of fiber, nutrients, and antioxidants, all essential for a healthy diet. These crops play a key role in the economy of many developed and developing countries and considerable efforts are being made to improve commercial traits using both conventional breeding and genetic engineering. However, growing social distrust in genetically modified (GM) crops in many countries has resulted in the adoption of very stringent and costly regulations disciplining the authorization of these crops, with the result that they have become very difficult or impossible to commercialize successfully (Sprink et al., 2016). Although genetic transformation of grape and apple crops has been used for the past two decades to enhance primarily biotic and abiotic tolerance, there are only a few examples of field evaluation or commercialization of transgenic plants worldwide (Kanchiswamy et al., 2015). A transition is needed toward more efficient and productive use of available genome sequences to meet growing demands for sustainable and safe intensification of food production. Here we explore the possibility of adopting next-generation plasmid-independent CRISPR/Cas9 genome editing approaches to develop improved grape and apple varieties that will probably avoid current GM regulations, and thus broaden the utility of this technology, with greater global acceptance levels. US Department of Agriculture (USDA) does not impose any GMO regulations to the plants with targeted mutagenesis generated by self-repair mechanisms, if they are free from *Agrobacterium* or any transgenic or foreign genetic materials; consequently, we assume there is high probability that CRISPR/Cas9 RNPs could be exempted from current GMO regulations (Waltz, 2012; Ledford, 2013; Jones, 2015). Nevertheless, the EU is uncertain to approve them and has yet to provide information on whether targeted mutation made by CRISPR/Cas9 or CRISPR/Cas9 RNPs fall outside regulatory criteria (Waltz, 2012; Jones, 2015).

CRISPR/Cas9 editing tools are efficiently exploited for gene mutation, repression, activation and epigenome editing in several model and crop plants, such as Arabidopsis, tobacco, rice, sorghum, maize, wheat, poplar, tomato, soya bean, petunia, citrus and recently grape and apple (Nishitani et al., 2016; Ren et al., 2016; Song et al., 2016). Meanwhile CRISPR/Cas9 RNPs DNA-free genome editing tools are successfully demonstrated in *Arabidopsis*, tobacco, lettuce, rice, petunia, and recently in wheat (Woo et al., 2015; Subburaj et al., 2016; Zhang et al., 2016).

To date, CRISPR/Cas9 RNPs editing tools have not been applied to genetic modification of grape and apple crops. Here, we demonstrate adoption of next-generation CRISPR/Cas9 RNPs technology for these fruit species to establish an efficient DNA-free method for the site-directed mutagenesis system. In the grapevine, PM (Gadoury et al., 2012; Pessina et al., 2016) is caused by the destructive fungal pathogen *Erysiphe necator*, an obligate biotroph infecting all green tissues and berries, resulting in drastic losses in yield and berry quality. Currently PM can be effectively controlled by frequent applications of fungicides in the field. However, the rapid emergence of new fungal strains and the hazardous effect of fungicides on the environment, combined with additional costs to growers (which can reach up to 20% of total production costs), demand the development of sustainable alternative strategies. Recently, it has been demonstrated that RNAi-mediated silencing of the susceptible gene (S-gene) *MLO-*7 significantly increases resistance to PM in the grapevine (Pessina et al., 2016). Here we targeted *MLO-7* for mutagenesis in order to increase resistance to PM in commercially important cultivar such as *Chardonnay.*

The enterobacterial phytopathogen *Erwinia amylovora* causes fire blight, an invasive disease that threatens the apple and a wide range of commercial and ornamental *Rosaceae* host plants. Although, many studies have identified candidate genes as suitable targets for increasing fire blight resistance via genetic engineering, currently no resistant cultivars are commercially available, due to the social and regulatory hurdles associated with GMO plants (Singh et al., 2006). Here we selected important fruit producing apple cultivar *Golden* delicious to target *DIPM-1, DIPM-2*, and *DIPM-4*, in order to increase resistance to fire blight disease (Meng et al., 2006). DIPM sequence structures are closely aligned with leucine-rich repeat receptor-like kinase receptors from several organisms, and furthermore DIPMs show direct physical interaction with the disease-specific (dsp) gene of *Erwinia amylovora*, which may act as a susceptible factor. Mutagenesis of *DIPM-1, 2, and 4* could provide apple cultivars resistant to fire blight disease.

We successfully show direct delivery of CRISPR/Cas9 RNPs to the grape and apple protoplast and efficient mutation of the targeted candidate genes. Targeted gene mutation, such as indel (insertion or deletion), was observed in 2 out of 2 specific sites of *MLO-7* in grapevine cultivars and single specific sites of each *DIPM-1, 2, and 4* in apple cultivars.

## Materials and Methods

### Grapevine Protoplast Preparation

Grapevine protoplast was isolated from 15 to 20 days old embryogenic calli and 15–20 days old *in vitro* micro propagated young and healthy leaves. Embryogenic calli or young and healthy leaves (10–15) were used for protoplast isolation and transformation. These plant materials were immersed in cell-wall digestion enzyme solution mix containing macerozyme R-10 (0.1–0.5%) and cellulase R-10 (1–2%) in 20 mM MES, 0.5 M mannitol, 20 mM KCL, and 10 mM CaCl_2_. Vacuum infiltration of plant materials took place with cell-wall digestion enzyme for 20 mins before incubating them at room temperature in a rotary shaker at 40 rpm for 4 h (embryogenic calli) or overnight (leaves). After digestion, protoplasts were filtered through Nylon mesh (75 μM), with the addition of 1:1 protoplast enzyme solution and W5 washing solution (5 mM glucose, 2 mM MES (pH 5.7), 154 mM NaCl, 125 mM CaCl_2_, and 5mM KCl), harvesting the protoplast by centrifugation at 100 × *g* for 5 mins. The supernatant was discarded and the protoplast re-suspended in 5 ml of W5 solution. A wide mouthed or point cut pipette tip was used to slowly transfer the protoplast to 5 ml of sucrose solution (21%), then centrifuged at 50 × *g* for 5 mins. A Pasteur pipette was used to suck the interface protoplast layer (viable and healthy protoplast), then re-suspended in 25 ml of W5 solution and incubated at 4°C for 1 h. This was centrifuged at 50 × *g* for 5 mins, the supernatant discarded and the protoplasts re-suspended in MMG solution (0.5 M mannitol, 4 mM MES (pH 5.7) and 15 mM MgCl_2_). The protoplast was counted using a hemocytometer and 2 × 10^5^ cells used for each CRISPR RNPs transformation. At least two biological replications and three technical replication sets were used to optimize and measure enzyme concentration and protoplast yield.

### Apple Protoplast Preparation

Apple protoplast was isolated from 20 to 25 days old *in vitro* micro propagated young and healthy leaves (10–15). The protocol for apple protoplast preparation was similar to that for the grapevine, except for the addition of hemicellulase to the cell-wall digestion enzyme solution (1–2%). The viability and density of grape and apple protoplast were determined using a haemocytometer, by staining the protoplast with fluorescein diactetate (FDA) as described elsewhere (Lei et al., 2015). At least two biological replications and three technical replication sets were used to optimize and measure enzyme concentration and protoplast yield.

### *In vitro* sgRNA Cleavage Assay

**FIGURE 1:**
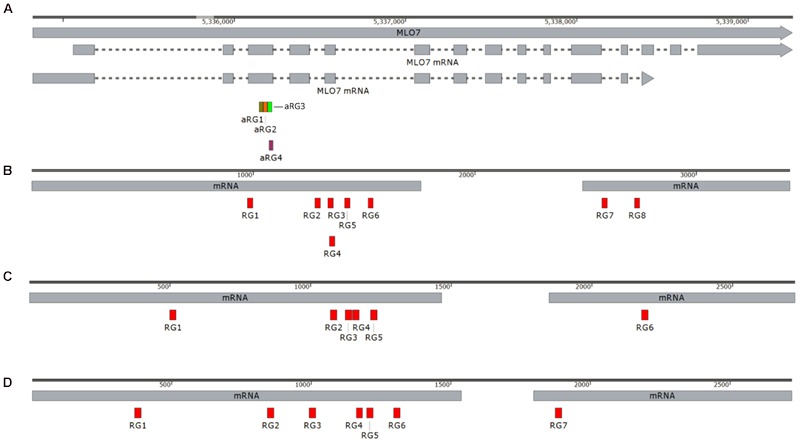
**(A)** Schematic diagram of the nucleotide sequence of the *MLO-7* gene locus of the grapevine with sgRNA target sites. **(B)** Schematic diagram of the nucleotide sequence of *DIPM 1.* **(C)** Schematic diagram of the nucleotide sequence of *DIPM 2*. **(D)** Schematic diagram of the nucleotide sequence of *DIPM 4*. Boxes with label under the 5′-3′ gray line indicate the position of each sgRNAs.

Commercially available ready to use recombinant Cas9 protein (160 kDa) and sgRNAs were purchased from ToolGen, Inc. (Seoul, Republic of Korea). The sgRNAs were designed for target-specific sites which have higher out-of-frame scores, to achieve maximum knock out efficiency in the *MLO-7* coding regions of the grapevine and the *DIPM1, DIPM 2* and *4* of the apple and highly efficient sgRNAs are selected via CRISPR RGEN Tools website^1^ (Bae et al., 2014; **Figure 1**; **Table 1**). For assessment of activity of CRISPR/Cas9 system, *in vitro* cleavage assay was performed as described elsewhere (Cho et al., 2013). Corresponding target sites were amplified by specific primer sets (**Table 2**), amplified PCR product (300 ng) is incubated for 60 min at 37°C with Cas9 protein (25 nM) and sgRNA (25 nM) in 10 μl NEB 3 buffer (1×). Reactions were stopped with 6× stop solution containing 30% glycerol, 1.2% SDS, and100 mM EDTA. Products were resolved with 1% agarose gel electrophoresis and visualized with EtBr staining (**Figure 3**). Purified recombinant Cas9 protein and sgRNA were used in a ratio of 1:3, 3:1, and 1:1 (in μg) to optimize the highest mutation efficiency during protoplast transformation. We used same amount of Cas9 protein for 1:3 and 1:1 conditions and three times more Cas9 protein for 3:1 condition.

**Table 1:** List of sgRNAs designed to target grape *MLO-7* gene locus and apple *DIPM-1, 2*, and *4* gene loci.

Target	sgRNA	Sequence (5′-3′)
MLO-7	RG1	TATGCATTTCTGAGAGTGTTGGG
	RG2	CACTTGGCACCCTTGTAAAAAGG
	RG3	CCAAAGATTTTAAGAACACATGC
	**RG4**	**TTTTAAGAACACATGCTCTGAGG**
DIPM-1	RG1	CGTCGTTTCAGCTCAACCCGGGG
	**RG2**	**CGATTGGCTGGTGAGGTAATTGG**
	RG3	AAGCACAGATCCCAGAACGGAGG
	RG4	GATCCCAGAACGGAGGGAGGTGG
	RG5	CGAGAGCAATTCCAAGGAGCGGG
	RG6	TAGCATTGGGACTACGTACAAGG
	RG7	CATGGATGAGGGACGACGATTGG
	RG8	TACTGGACTTGCGAATCTCGGGG
DIPM-2	RG1	ACGGAAGAGAGAGGTTGCGGAGG
	RG2	CAGTTTCTAGAAGCTTCGAGCGG
	RG3	CTCGTACCCGGGTTGGGCAGAGG
	**RG4**	**GGTCGGATGGTGTTCTTCGAAGG**
	RG5	CCCGCCTTTCCCCAGCATCTCGG
	RG6	CACGGGGAAGTGTCCCTCCGTGG
DIPM-4	RG1	AGCGACTACGCGGTCTTTATTGG
	RG2	GAACAAGCCGGCAAGGGGCTCGG
	RG3	ACGATCGCAACGATTGCGCCGGG
	RG4	AAGGTGTACGCGAACAGCGGGGG
	RG5	ACGGTACAAACGCGACGGACAGG
	RG6	GGGGAAAGGAAGCCTAGGGACGG
	**RG7**	**GCTGTATTCCGCATGAATCCTGG**

*Sequences of sgRNA in bold indicate selected sgRNAs used in the study for transformation. Underlined nucleotides indicate PAM motifs in sgRNA sequences.*

**Table 2:** List of primers used for *in vitro* cleavage assay and targeted deep sequencing.

Target	Sequence (5′-3′)
MLO-7 *in vitro* cleavage assay	F; GCAGTGGTTAAAAGGCAGAC R; CTTGGTTCTTCCCAAAGCC
MLO-7 deep sequencing	F; CCAAAGGTCTAACCCTTTTC R; GGGAAACACCTTTTTCAGTC
DIPM-1 *in vitro* cleavage assay	For RG1 – RG6 F; TTTTAATCTTCAACGTCTCC R; TTGCCTGAAAATAAGCCCTC
	For RG7 and RG8 F; TAGTAACCAAAGGGAAGTGG R; TTCAACACTTGCCACATTGC
DIPM-1 deep sequencing	F; GTCTTATGCCTCTTTGCGG R; CTCCAGACTGTATAGCTGAG
DIPM-2 *in vitro* cleavage assay	For RG1 F; TGCACAAATAACCGAGTCTC R; ACTATGATGGCGATTAGAGC
	For RG2 to RG5 F; CAAGGGTACATCAAACGACC R; AAATTTACCGTGGAGAACCC
	For RG6 F; ACATCCTCTTAGACAAGACC R; AGCACAAACGAAAACGAGAG
DIPM-2 deep sequencing	F; CGCTGCTCCTGTACTGCTAC R; GTCGAGCACCGCCTTGTA
DIPM-4 *in vitro* cleavage assay	For RG1 F; AGAAAAACAAGCCTTTCGCG R; TCCGTACAATTCGTTGTTGG
	For RG2 to RG6 F; CACCAACAACGAATTGTACGG R; AGTAATAAGCACTCAGCCTC
	For RG7 F; TGCAGTTTGAGTCTAATGCG R; CCTCAATGTTCTTGTACCTC
DIPM-4 deep sequencing	F; GATGTAATTAAGGGAATCGG R; CAATCTTGCAATGGCGTGAA

### Protoplast Transformation with CRISPR RNPs

In order to optimize efficient targeted mutagenesis of grapevine *MLO-7* and the *DIPM-1, 2*, and *4* of apple gene loci, 2 × 10^5^ re-suspended protoplasts were transformed with Cas9 protein and sgRNA in a ratio of 1:3, 3:1, and 1:1 (Woo et al., 2015; Subburaj et al., 2016). Protoplast volume 200 μl (2 × 10^5^ cells) and RNPs for example 3:1 is Cas9 90 μg (stock 10 μg/μl) sgRNA 30 μg (stock 10 μg/μl) is used for transformation. Prior to the transformation, Cas9 and sgRNA were pre-mixed and incubated at room temperature for 10 mins. The protoplast, Cas9 and sgRNA mix were mixed and an equal volume of PEG 4000 added, gently but immediately mixing the tube before aggregation occurred and incubating it for 20 mins at room temperature. Four hundred microliter or an equal volume of W5 solution were added, mixed and incubated at room temperature for further 10 mins. An additional 800 μl were added or the volume of W5 solution doubled, mixed and incubated at room temperature for further 10 mins. This was centrifuged at 50 × *g* for 5 mins, the supernatant discarded and 1 ml of W5 solution added, followed by incubation at room temperature in the dark overnight. The lower sediments were collected for genomic DNA isolation. Three biological replications are performed for protoplast transformation and isolated genomic DNA from protoplast transfected cells are further used for targeted deep sequencing.

### Targeted Deep Sequencing

Sequence at the sgRNA target sites were analyzed as described elsewhere (Woo et al., 2015). Corresponding target sites were PCR amplified using the primers listed in **Table 2**. Amplifications were performed using Phusion polymerase. Amplified PCR products were sequenced using the Illumina MiSeq platform (Quail et al., 2012). Mutations induced by CRISPR RNPs were calculated based on the indels around the CRISPR RNPs cleavage sites (3 bp upstream of PAM) using CRISPR RGEN Tools software^2^. Three biological replications are performed for targeted deep sequencing. Average of three biological replications are used for statistical analysis to determine percentage of indel ratio.

## Results

### Protoplast Isolation in Grapevine and Apple Cultivar

In the grapevine, embryogenic calli provided a higher yield of up to 3.6 × 10^6^ with 90% viability when using 1.5% cellulase R-10 and 0.4% macerozyme R-10, with 20 min of vacuum infiltration followed by 3 h incubation with gentle shaking (**Table 3**). Conversely, leaves gave a lower protoplast yield and lower viability, with incubation periods of up to 24 h. In the apple, cell wall digestion with 1.5% cellulase R-10, 0.4% macerozyme R-10, and 1% hemicellulase provided a maximum yield of 1.0 × 10^6^ with 80% viability, with 20 min of vacuum infiltration followed by 24 h incubation with gentle shaking, compared to the other ranges of various cell wall digesting enzyme concentrations (**Table 1**). In the apple, we selected leaves and avoided callus explants, due to their hard structure and lower protoplast yield.

**Table 3:** Grape and apple protoplast yield with various concentrations of the cell-wall digestion enzymes from leaves (10-15 healthy leaves) and embryogenic calli (100 mg).

Plant materials	Cultivar	Enzyme concentration	Protoplast yield
**Grapevine cultivar**			
Leaves	Chardonnay	Macerozyme R10–0.1%, Cellulase R10–1%	2 × 10^6^ protoplast per ml
Leaves	Chardonnay	Macerozyme R10–0.15%, Cellulase R10 – 1%	2 × 10^5^ protoplast per ml
Embryogenic calli	Chardonnay	Macerozyme R10–0.1%, Cellulase R10–1%	4 × 10^6^ protoplast per ml
Embryogenic calli	Chardonnay	Macerozyme R10–0.15%, Cellulase R10–1%	3 × 10^6^ protoplast per ml
**Apple cultivar**			
Leaves	*Golden delicious*	Macerozyme R10–0.15%, Cellulase R10–1%, Hemicellulase–1%	1.8 × 10^6^ protoplast per ml
Leaves	*Golden delicious*	Macerozyme R10–0.15%, Cellulase R10–1%, Hemicellulase–1.5 %	1.0 × 10^6^ protoplast per ml

*At least two biological replications and three technical replication sets were used to optimize and measure enzyme concentration and protoplast yield.*

### Targeted Mutagenesis of the Grapevine and the Apple Using CRISPR/Cas9 RNPs

**FIGURE 2:**
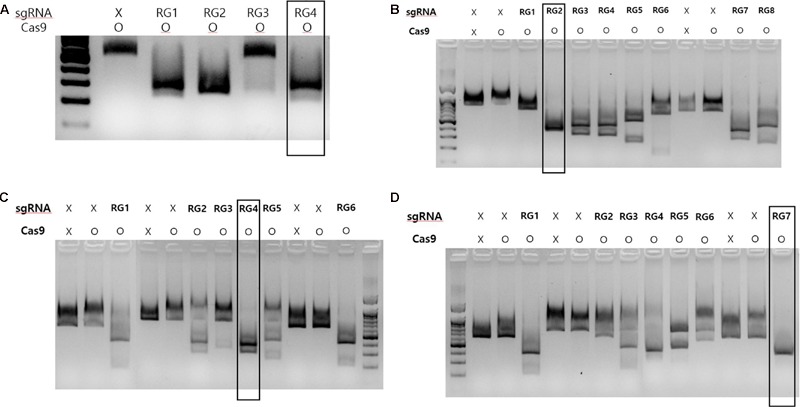
**CRISPR/Cas9 RNPs *in vitro* digestion assay results of each sgRNAs used in the study. (A)** *In vitro* digestion of targeted loci at MLO-7 gene. **(B)** *In vitro* digestion of targeted locus in the DIPM-2 gene. **(C)** *In vitro* digestion of targeted locus in the DIPM-2 gene. **(D)** *In vitro* digestion of targeted locus in the DIPM-4 gene. In each group, non-treated (sgRNA: x; Cas9: x) or Cas9-only (sgRNA: x; Cas9: o) samples were used as a control. After treatment with Cas9 with targeted sgRNA (RG1 to RG8 in each group) amplified target genomic DNA was digested and smaller bands were detected in gels after electrophoresis. Groups showing an intense band after digestion (indicated with black boxes) were selected and used for further experiments.

To identify suitable sgRNAs for targeted mutagenesis, we designed several sgRNAs for each gene used in the present study and then cleavage frequencies of each sgRNAs were assessed using an in vitro digestion assay. All the sgRNAs used in this study were designed to pair with their corresponding 20 nucleotide target sites in *MLO-7, DIPM-1, 2* and *4* gene loci and to assist Cas9 to create site-specific double strand breaks (DSBs) at 3 bp upstream of the PAM motifs (**Table 1**). As shown in **Figure 2**, sgRNAs in each gene showing the highest cleavage rate were selected for further study. To target the grapevine *MLO-7* gene locus, we used sgRNA RG4 (**Figure 2**). Similarly, in the apple we used three specific sgRNAs for the *DIPM-1* (RG2), *DIPM-2* (RG4), and *DIPM-4* (RG7) loci (**Figure 2**), respectively.

### Targeted Deep Sequencing to Analyze the Mutation Efficiency of CRISPR/Cas9 RNPs

In order to detect the mutation efficiency and mutation patterns at different sites in the grape gene locus *MLO-7* and the apple gene loci *DIPM-1, 2*, and *4*, we employed targeted deep sequencing of genomic DNA obtained from each protoplast pool during PCR amplification. Total genomic DNA was extracted from transformed protoplast, while CRISPR/Cas9 target sites in *MLO-7, DIPM-1, 2*, and *4* were amplified using site-specific primers (**Table 2**). PCR amplified products were subjected to targeted deep sequencing. Targeted deep sequencing results showed that there were various number indel mutation frequencies (%) for each CRISPR sgRNA sample (**Table 4**).

**Table 4:** Mutation rate assay by targeted deep sequencing in *MLO-7, DIPM 1, 2*, and *4.*

Target gene	Sample name	Number of Reads (more than minimum frequency)			Number of insertion mutations			Number of deletion mutations			Indel ratio (average, %)
		1	2	3	1	2	3	1	2	3	
MLO-7 (RG4, grape)	sgRNA only	56302	52455	54565	0	0	0	0	0	0	0.00
	Cas9 only	9924	10123	10001	0	0	0	0	0	0	0.00
	Cas9: sgRNA, 1:1	51558	52015	52206	0	0	0	49	55	64	0.10
	Cas9; sgRNA, 1:3	56546	55432	56421	2	4	6	71	74	69	0.10
	Cas9; sgRNA, 3:1	67286	64532	66876	42	57	68	10	12	9	0.10
DIPM1 (RG2, apple)	sgRNA only	58020	57987	58911	0	0	0	2	0	1	0.00
	Cas9 only	53727	54455	55432	0	0	0	5	7	8	0.00
	Cas9: sgRNA, 1:1	60903	60467	60787	0	0	0	16	22	13	0.00
	Cas9; sgRNA, 1:3	65674	64678	65632	0	0	0	15	19	22	0.00
	Cas9; sgRNA, 3:1	61565	62639	60166	0	0	0	4150	4500	3989	6.70
DIPM2 (RG4, apple)	sgRNA only	22397	22565	22001	0	0	0	0	0	0	0.00
	Cas9 only	16021	17089	17345	0	0	0	0	0	0	0.00
	Cas9: sgRNA, 1:1	17847	18945	17923	447	565	472	140	167	178	3.30
	Cas9; sgRNA, 1:3	17965	17456	17989	80	74	92	2	4	5	0.50
	Cas9; sgRNA, 3:1	17005	17233	17565	291	300	267	271	288	298	3.30
DIPM4 (RG7, apple)	sgRNA only	20239	20679	20899	0	0	0	0	0	0	0.00
	Cas9 only	32096	32198	32345	0	0	0	0	0	0	0.00
	Cas9: sgRNA, 1:1	42871	43211	43001	691	701	719	2253	2300	2310	6.90
	Cas9; sgRNA, 1:3	22055	22100	22189	0	0	0	555	567	590	2.50
	Cas9; sgRNA, 3:1	30240	31000	30319	0	0	0	1835	2000	2187	6.10

As shown in **Table 4**, various mutation patterns including indels were detected in all the different sgRNA RNPs complex transformed protoplast samples, whereas no mutations were detected in sgRNA-only or Cas9-only transformed protoplast samples. These results demonstrate direct delivery of CRISPR RNPs to grapevine and apple protoplast, and indel mutagenesis efficiency of 0.1% and 0.5 to 6.9% for targeted distinct sites of endogenous *MLO-7 and DIPM-1, 2*, and *4 via* DSBs, respectively.

## Discussion

Plant protoplasts constitute a dynamic and versatile system for CRISPR/cas9 genome editing in plants and has been widely adopted in several crop species for functional analysis of the traits concerned, cellular localization, and studies of multiple signaling cascades (Shan et al., 2013; Xie and Yang, 2013; Zhao et al., 2016). CRISPR/Cas9 or other genome editing tools mediated protoplast transfection system has been successfully adopted in *Arabidopsis*, rice, wheat, maize, tobacco, lettuce, and petunia (Jiang et al., 2013; Li et al., 2013; Shan et al., 2013; Wang et al., 2014; Gao et al., 2015; Woo et al., 2015; Subburaj et al., 2016), however, a similar system has not been developed for the grapevine and apple. In this study, we isolated protoplast from embryogenic calli and leaves of grapevine and apple cultivar in order to standardize an efficient protocol for the transient expression system of CRISPR/Cas9 RNPs. Protoplast isolation, transfection and transient gene expression system in grape and apple has been little explored and most of the available methods have not been updated for two decades (Doughty and Power, 1988; Patat-Ochatt et al., 1993; Patat-Ochatt, 1994; Reustle et al., 1995; Zhu et al., 1997; Saito and Suzuki, 1999; Fontes et al., 2010). Protoplast viability, yield, and efficient transfection depend on various factors, such as the concentration of cell wall digestion enzymes, buffer conditions, the osmotic status of protoplasts, the incubation period, and the type of explants used for protoplast isolation. In the current study, all these variables were updated and optimized in order to achieve a better yield. This is the first report of successful demonstration of CRISPR/Cas9 RNPs mediated protoplast transformation in grapevine and apple cultivars. Our method for transient expression of genome editing tools in the protoplast to target the gene of interest with specificity and higher efficiency should help grapevine and apple scientists to analyze the traits concerned in the host plant within a day or two. Furthermore, future work on regeneration of genome edited protoplast will provide an opportunity to develop DNA-free genome edited grapevine and apple fruit crop plants. One such example is regeneration of apple plants from meristem derived callus protoplast (Saito and Suzuki, 1999).

**FIGURE 3:**
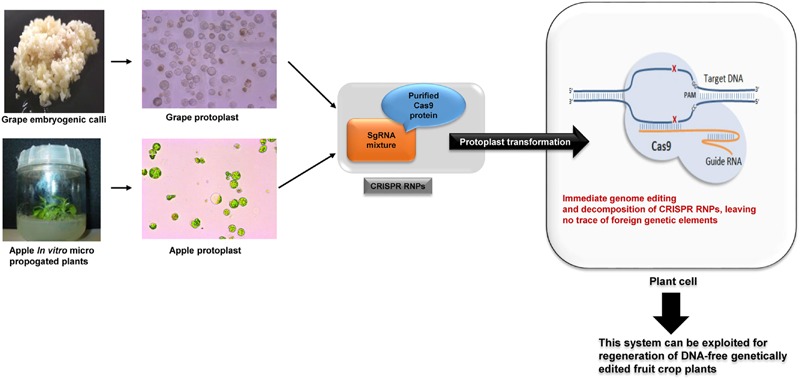
**Schematic diagram of CRISPR RNPs direct delivery to grape and apple plant cells to produce DNA-free genetically edited crop plants**.

CRISPR/Cas9 is easy to prepare, scalable and affordable compared to ZFNs and TALENs. However, the broader application of plasmid mediated CRISPR/Cas9 to life sciences, biotechnology and medicine is limited by off-target effects, unwanted integration of plasmid vectors into the genome and possible GMO regulations (Hendel et al., 2015; Zhang et al., 2015). In order to overcome these limitations, we delivered CRISPR/Cas9 RNPs rather than plasmids directly into the protoplast cells and showed that RNPs enable efficient genome editing, while avoiding unwanted integration of plasmid DNA in the host genome, similar to other recent studies done in human, animal and plant cells (**Figure 3**; Kim et al., 2014; Lee et al., 2014; Liu et al., 2015; Woo et al., 2015; Subburaj et al., 2016).

Recently several groups have demonstrated that CRISPR/Cas9 can induce unwanted mutations in off-target sites, differing from on-target sites by up to 5 nt, leading to questions about their specificity (Cradick et al., 2013; Fu et al., 2013; Hsu et al., 2013; Tan et al., 2015). We and others have proposed various strategies to improve the on-target specificity of CRISPR/Cas9 (Koo et al., 2015; Kanchiswamy et al., 2016). Synthesis of unique sgRNA (Cho et al., 2014; Fu et al., 2014; Sung et al., 2014), Cas9 nickases and web based computer programs to identify unique target sites (Mali et al., 2013; Ran et al., 2013; Bae et al., 2014) and CRISPR/Cas9 RNPs (Woo et al., 2015). In order to facilitate the highest site-specific mutation frequency in grape and apple protoplast, we titrated the ratio of Cas9 and sgRNAs, in a similar way to our previous studies (Hsu et al., 2013; Woo et al., 2015). In this study, we employed three different Cas9: sgRNA ratios, i.e., 3:1, 1:1, and 1:3, for protoplast PEG mediated transformation in the grape and the apple. We determined that the 3:1 ratio for MLO-7 in the grapevine, the 3:1 ratio for DIPM 1, the 1:1 and 3:1 ratio for DIPM 2 and the 1:1 ratio for DIPM 4 in the apple resulted in highest mutation frequency. Here, we showed the critical advantage over plasmid mediated genome editing delivery by titrating the Cas9:sgRNA ratio to achieve maximum mutation frequency (Liu et al., 2015; Kanchiswamy et al., 2016).

This study demonstrated direct delivery of CRISPR/Cas9 RNPs to grape and apple protoplasts and site-directed mutation of the grape gene locus *MLO-7* and the apple gene loci *DIPM-1, 2*, and *4*.

## Conclusion

We demonstrated efficient targeted mutagenesis in the grapevine gene locus *MLO-7* and the apple gene loci *DIPM-1, 2*, and *4*, using direct delivery of CRISPR RNPs. Although the mutation efficiency was found to vary with the targeted gene locus and the ratio of Cas9 and sgRNA, mutation patterns and frequency assays showed CRISPR RNPs to be an effective strategy for targeted mutagenesis of gene loci in grape and apple protoplasts. This method has already shown improved features compared to plasmid-mediated genome editing in humans, animals and plants, such as higher efficiency, significantly reduced off-target effects and more rapid editing activity after delivery (Liu et al., 2015; Woo et al., 2015; Kanchiswamy, 2016; Kanchiswamy et al., 2016). Furthermore, in plants, the new varieties obtained with this approach may be deregulated from current GMO legislations, as the Cas9 protein-guide RNA complexes will rapidly decompose in regenerating cell cultures. Further studies are now required to optimize plant regeneration from CRISPR RNPs transformed protoplast to explore the applications of this technology at field level.

## Author Contributions

CN, O-JK, and M-HJ performed and designed experiments. RV, SK, J-SK, RiV, and MM provided materials, funding, plan for the experiment. CN wrote manuscript with the cooperation and support of all co-authors.

## Conflict of Interest Statement

The authors declare that the research was conducted in the absence of any commercial or financial relationships that could be construed as a potential conflict of interest.

## Footnotes

1 http://rgenome.net/

2 http://rgenome.net/
